# Intermittent theta burst stimulation with synchronised transcranial alternating current stimulation leads to enhanced frontal theta oscillations and a positive shift in emotional bias

**DOI:** 10.1162/imag_a_00073

**Published:** 2024-01-25

**Authors:** Paul M Briley, Clement Boutry, Lucy Webster, Domenica Veniero, Catherine Harvey-Seutcheu, JeYoung Jung, Peter F Liddle, Richard Morriss

**Affiliations:** Mental Health and Clinical Neurosciences, School of Medicine, University of Nottingham, Nottingham, United Kingdom; Nottingham National Institute for Health and Care Research (NIHR) Biomedical Research Centre, Nottingham, United Kingdom; Institute of Mental Health, Nottinghamshire Healthcare NHS Foundation Trust, Nottingham, United Kingdom; NIHR Applied Research Collaboration East Midlands, Nottingham, United Kingdom; School of Psychology, University of Nottingham, Nottingham, United Kingdom; NIHR MindTech MedTech Collaborative, University of Nottingham, Nottingham, United Kingdom

**Keywords:** theta oscillations, emotional bias, transcranial magnetic stimulation, TMS, transcranial alternating current stimulation, tACS

## Abstract

Repetitive transcranial magnetic stimulation (rTMS), delivered to left dorsolateral prefrontal cortex, is an FDA-approved, and NICE-recommended, neuromodulation therapy for major depressive disorder (MDD). However, there is considerable inter-individual variability in rate and extent of clinical response, leading to a focus on approaches for optimising its effectiveness. We present findings from a non-patient study evaluating an approach that combines an efficient type of rTMS—“intermittent theta burst stimulation” (iTBS)—with a second neuromodulation technique—“transcranial alternating current stimulation” (tACS). tACS is delivered in synchrony with the iTBS with the intent of optimising the brain state during stimulation. In four separate sessions, we delivered 3 minutes of iTBS+tACS, iTBS+sham, sham+tACS, or double sham. We measured changes from pre- to post-stimulation in brain theta (4–8 Hz) oscillatory activity using electroencephalography, and we measured emotional bias post-stimulation using a well-studied emotion identification task. Theta activity has previously shown relationships with response to rTMS, and emotional bias has been proposed as a marker of potential antidepressant efficacy. We found that frontal theta power was enhanced following the dual therapy, building up over the 15-minute post-stimulation period to exceed that following either stimulation technique alone or double sham. Emotional bias, measured 20 minutes post-stimulation, was also significantly more positive following dual therapy. These findings indicate that tACS-synchronised iTBS (tsiTBS) holds promise as an augmentation approach for rTMS, which awaits validation in multi-session patient studies.

## 1 Introduction

Repetitive transcranial magnetic stimulation (rTMS) is a minimally invasive neuromodulation therapy that is FDA-approved for the treatment of major depressive disorder (MDD). rTMS delivers magnetic pulses via a coil placed over the scalp, temporarily modulating the excitability of the underlying cortex, leading to neuroplastic changes in and between connected brain regions (To et al., 2018). Most commonly, rTMS is delivered over the left dorsolateral prefrontal cortex (DLFPC), a key component of the brain’s “executive control network” (ECN) of regions involved in decision-making and working memory (Dosenbach et al., 2008). Clinical response, and rate of response, to rTMS remains variable. In a large clinical trial for treatment-resistant MDD, which delivered 20–30, once-daily, rTMS sessions targeting left DLPFC, the observer-rated depressive symptoms of 20% of patients improved rapidly across the first week (the ideal outcome), whilst 10% did not improve, and the remainder improved gradually over several weeks with benefit appearing to plateau (Kaster et al., 2019). Given the overall efficacy, and high acceptability (H. Li et al., 2021), of rTMS, methods to augment its effectiveness that are easily implemented in the clinical setting will be valuable. We are developing one such approach, by combining rTMS with a second form of neuromodulation technique, transcranial alternating current stimulation (tACS).

Whilst rTMS delivers discrete magnetic pulses using a coil over the scalp, tACS delivers an oscillating waveform between two scalp electrodes, mimicking brain oscillatory activity (Antal & Paulus, 2013). Oscillatory activity, a manifestation of neural circuit dynamics and recordable using non-invasive electroencephalography (EEG), underlies brain connectivity and excitability (Von Stein & Sarnthein, 2000; Whitman et al., 2013). The amplitude, phase, and location of oscillations thus represent important aspects of “brain state.” One of the key reasons for variability in the effects of rTMS (which applies to its basic physiological effects, as well as its clinical effects (López-Alonso et al., 2014)) is thought to be differences in brain state at the time of stimulation (Huang et al., 2017). tACS may be able to modify a key aspect of brain state (endogenous oscillatory activity) relevant to the effects of rTMS.

There is evidence that tACS can modulate oscillatory power, although these effects may be dependent on a precise match between the stimulation frequency and the natural oscillatory frequencies in an individual; rTMS itself can lead to short-lived modulation of oscillatory activity (Herrmann et al., 2016; Veniero et al., 2015). Hosseinian, Yavari, Biagi, et al. (2021) observed more robust changes by combining 8 minutes of “alpha” (10 Hz) frequency rTMS with synchronised alpha tACS (both delivered bilaterally to DLPFC). Alpha rTMS to left DLPFC is the traditional stimulation approach used for treating MDD, although the typical clinical protocol takes around 30 minutes per session. A newer rTMS protocol used for treating MDD, called “intermittent theta burst stimulation” (iTBS), shows equivalent clinical response (Blumberger et al., 2018), but takes only 3 minutes. Whilst traditional rTMS delivers trains of TMS pulses at a single frequency, iTBS delivers cycles of 50 Hz pulse triplets with triplets repeating at 5 Hz—a “theta” frequency—with 2 s on-time and 8 s off-time (Huang et al., 2005).

iTBS could be delivered in synchrony with theta-frequency tACS, with TMS pulse triplets occurring around the tACS peaks. Theta frequencies are of further interest, since theta brain oscillations (4–8 Hz) are integral to the functioning of the ECN (Kawasaki et al., 2010; Mizuhara & Yamaguchi, 2007; Sauseng et al., 2005, 2007) of which the usual target of clinical TMS, the DLPFC, is a critical part. Frontal theta power measured during a working memory task has been found to predict response to rTMS for MDD (Bailey et al., 2018; Klooster et al., 2023). Thus, enhancing theta during stimulation might improve rTMS efficacy. Indeed, Li et al. (2016) found that using an appropriate cognitive task to enhance frontal theta power prior to rTMS led to a better antidepressant response than when a control task was used. Such tasks may not be suitable for people with more severe depression due to associated cognitive and motivational difficulties in MDD (Austin et al., 2013; McDermott & Ebmeier, 2009). Synchronised theta-frequency tACS may be able to improve TMS efficacy without a task, by contributing to a more efficacious high frontal theta brain state and providing an optimal neural oscillatory context for iTBS.

That theta power can be robustly enhanced using neuromodulation approaches was later demonstrated by Hosseinian, Yavari, Kuo et al. (2021), who delivered 20 minutes of 6-Hz tACS and synchronised traditional 6 Hz (non-iTBS, theta-frequency) rTMS. Theta power measured during an n-back working memory task was enhanced after the combination, or 6-Hz tACS alone, although not after rTMS alone. Theta during a working memory task was only measured immediately after stimulation. Resting theta remained elevated for around 50 minutes post-stimulation and was greatest after combined stimulation. Most recently, Maiella et al. (2022) quantified changes in resting-state theta and high-frequency “gamma” power immediately after iTBS with synchronised theta-tACS, gamma-tACS, or sham-tACS. They found a change (increase) only in gamma power and with gamma tACS. Given concerns that gamma oscillations may not be well-placed to serve a role beyond regulating local cortical excitatory-inhibitory balance (Merker, 2013; Ray & Maunsell, 2015), exploring the ability of tACS in combination with iTBS to enhance theta oscillations remains an important avenue of research.

We report here measurements of theta oscillatory power, during rest and working memory tasks, at multiple time points, following either iTBS with synchronised theta-tACS, iTBS with sham tACS, sham iTBS with theta-tACS, or double sham. We term the dual therapy, “tACS-synchronised iTBS” (tsiTBS). Our measurements were conducted in healthy volunteers. We predicted that tsiTBS would lead to greater enhancement of theta power than TMS alone, thus indicating induction of a potentially more efficacious high theta brain state. To ascertain whether the dual approach might hold promise for the treatment of MDD, we obtained measurements of “emotional bias” (judgements of emotions expressed in ambiguous face stimuli) following each of the four stimulation types. MDD is characterised by biases towards perceiving negative emotions (Delle-Vigne et al., 2014; Gollan et al., 2008). There is evidence for normalisation of automatic negative biases following successful antidepressant treatment (Ruohonen et al., 2020). Changes in emotional bias have been observed following single doses of antidepressants, including in healthy volunteers (Bhagwagar et al., 2004; Harmer et al., 2003), so it is conceivable that such shifts may be observed following single sessions of neuromodulation approaches. We predicted that the combination treatment would be associated with more positive emotional bias measured post-stimulation than TMS alone.

## 2 Methods

### 2.1 Overview of study

**Fig. 1.**
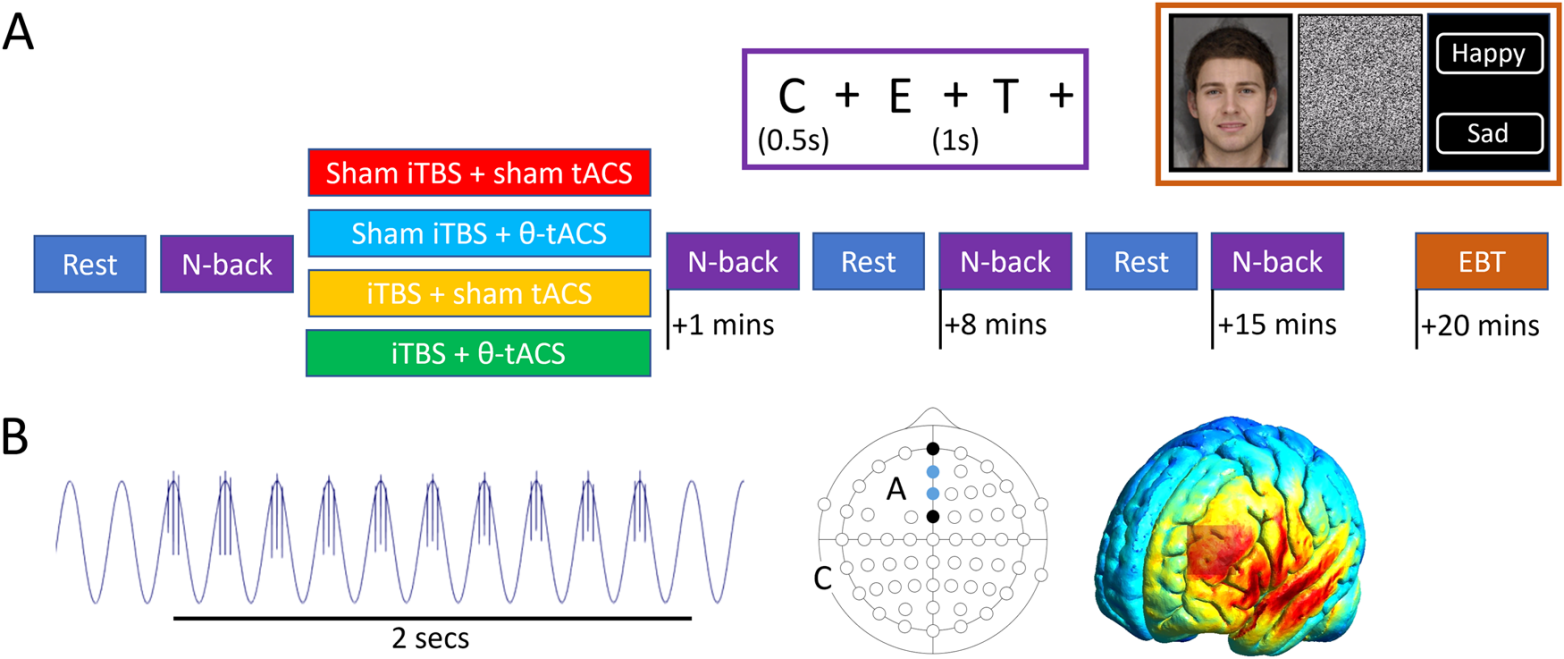
Structure of an experimental session and illustration of the stimulation approach. (A) EEG was recorded during rest (eyes closed and open) and a two-back working memory task before, and at multiple time points after, one of four types of stimulation. The emotional bias tasks (EBT) were commenced 20 minutes post-stimulation. Insets illustrate the n-back task (purple border) and the EBT task (orange border; CANTAB® [Cognitive assessment software]. Cambridge Cognition (2023). All rights reserved. https://cambridgecognition.com/). (B) On the left is a segment of the tACS waveform, recorded near the tACS anode, with TMS pulses visible as vertical bars. The positions of the anode (“A,” at 10–20 location F3) and cathode (“C,” at TP9), as well as the positions of the electrodes used to calculate theta power (blue circles, AFz and Fz), and the positions of the ground and reference electrodes (black circles, FPz and FCz), are shown in the middle. On the right is an illustration of the associated electric field at the anodal peaks (computed in simNIBS including electrode size and position with the MNI152 model, visualised in Gmsh).

Participants completed up to four experimental sessions, each at least 5 days apart. Each session followed the same layout, and sessions differed by the type of stimulation received (tsiTBS, iTBS+sham, sham+tACS, double sham). The order of stimulation conditions for each participant was selected sequentially from a randomised list. At the start of each session, participants completed a short, 60-trial, practice version of the n-back working memory task and EEG; tACS and TMS were set up. Participants sat in an electrically shielded room. EEG was recorded during resting-state (eyes closed and open) and the n-back task, with room lights switched off (Fig. 1A). The experimenters then entered and delivered the stimulation, after which they left and further runs of n-back (1-, 8-, and 15-minutes post-stimulation) and resting-state (5- and 12-minutes post-stimulation) were obtained. Lights were switched on, the EEG cap and tACS electrodes were removed, and participants completed two variants of the Cambridge Cognition CANTAB emotional bias task (20 minutes post-stimulation; https://cambridgecognition.com/). They then completed a side-effects questionnaire.

### 2.2 Participants

All participants were aged 18–39 years old. Exclusion criteria included: currently under the care of mental health services; current or past diagnosis of bipolar or psychotic disorder, substance abuse or dependence, dementia or mild cognitive impairment; PHQ-9 scores of 10 or greater; suicidal ideation or behaviour within the past 3 months; and current or prior neurological conditions. The study was approved by the Research Ethics Committee of the University of Nottingham Faculty of Medicine and Health Sciences (Ethics Reference Number FMHS 28-0622), and written informed consent was obtained from all participants. Participants were compensated with a shopping voucher for each session completed.

We tested 26 participants in total (16 female, mean age ± SD 23.6 ± 4.7 years, 10 reported an ethnicity other than white). Two were classed as ambidextrous and the remainder as right-handed by the criteria of Oldfield (1971). The majority (N = 18) were university students. Mean (±SD) PHQ-9, and GAD-7, scores prior to the first session were 3.3 (±2.2), and 2.5 (±2.6), respectively. Thirteen participants provided data for all four conditions, eight for three conditions, one for two conditions, and four for one condition. For the first two participants, the tACS-only condition was not included. In addition, those participants completed a three-back version of the n-back task, rather than a two-back version. Only their emotional bias task data were included, therefore (the protocol was modified following the first two participants to use the two-back task, given the reported difficulty of the three-back task, and to add the tACS-only condition). Other reasons for not completing all sessions were: participant unable to make time for further sessions (due to examinations, holidays, or university course finishing, N = 4), failure of the EEG system towards the end of the experiment (failure unrelated to current study, N = 4), and no reason given (N = 3). Number of participants included for each condition were as follows: tsiTBS = 21 (23 for emotional bias tasks), iTBS+sham = 19 (21), sham+θ-tACS = 18 (18), and double sham = 18 (20). Seven participants completed the tsiTBS condition first (27% of all first sessions), seven completed it second (32%), five completed it third (24%), and four completed it last (31%).

### 2.3 Neuromodulation

#### 2.3.1 Transcranial magnetic stimulation (TMS)

TMS was delivered using a Magstim Rapid2 Plus with a D70 Alpha Flat Coil. Participants wore foam earplugs to attenuate noise throughout stimulation. Passive resting motor threshold (rMT) was obtained at the start of the first stimulation session. rMT was defined as the lowest TMS intensity required to elicit a twitch of the right thumb on 50% of trials, estimated with an adaptive PEST algorithm (Borckardt et al., 2006) and stimulation in the vicinity of the “C3” 10–20 electrode position.

Stimulation consisted of 600 pulses of iTBS (3.3 minutes) delivered at 80% rMT intensity with standard parameters (Huang et al., 2005): 20 cycles with 2-/8-second on-/off-time, pulse triplets (20-ms inter-pulse interval) repeating at 5 Hz. The coil was placed tangential to the scalp over F3, with handle pointed backwards 45° to the midline. F3 is commonly used as the approximate location of left DLPFC (Herwig et al., 2003). Similar to Hosseinian, Yavari, Biagi et al. (2021) and Hosseinian, Yavari, Kuo, et al. (2021), the positioning of both the tACS and TMS at F3 added approximately 4 mm to the coil-cortex distance. Stokes et al. (2007) estimate than an additional millimetre of separation equates to an increase of 2.8% absolute stimulator output required for rMT. As in Hosseinian et al., we did not increase the stimulator intensity to compensate for this (doing so significantly impacted the tolerability of stimulation). Ma et al. (2023), combining data from several studies, report a relationship between “active” motor threshold (aMT) and rMT of: $aMT=1.062\cdot \;rMT^{0.92}$. Using this formula and applying the distance correction of Stokes et al. to our delivered iTBS intensity values, suggests that our iTBS would be received at approximately 80% of aMT (±2%). This is likely greater than in Hosseinian et al., who delivered stimulation at 70% or 80% of aMT. The relationship between motor threshold and optimal stimulation intensity of left DLPFC is unclear. Traditionally, iTBS has been delivered at 80% of aMT, but more recently, 80% of rMT has been used (Chu et al., 2021).

In conditions with active tACS, the onset of each iTBS cycle was triggered by the tACS system (via a BNC cable delivering a pulse to the Magstim Stimulator Interface Module). In conditions with sham tACS, the onset of the full set of 20 iTBS cycles was triggered. In either case, an identical course of iTBS was delivered. Sham iTBS used a perpendicular vertical coil rotation, with coil wing pressed onto the head to elicit pressure, vibration, and acoustic stimulation.

#### 2.3.2 Transcranial alternating current stimulation (tACS)

tACS was delivered using a Nurostym tES system (Neuro Device Group S.A., Poland) with 9 cm^2^ square carbon silicone rubber electrodes placed at F3 (anode) and TP9 (cathode)—the same locations used by Hosseinian, Yavari, Biagi et al. (2021) and Hosseinian, Yavari, Kuo, et al. (2021) to target left DLPFC (they used a bilateral montage to also target right DLPFC). Electrodes were placed prior to fitting the EEG cap by using the Beam F3 algorithm (Beam et al., 2009) to site the anode and by placing the cathode behind the left ear. Impedance was kept below 15 kOhms. During stimulation, a 5-Hz alternating current with 1 mA peak-to-peak amplitude was applied. It ramped on over 10 seconds, after which the Nurostym device sent pulses to trigger each iTBS cycle (Fig. 1B). In active tACS conditions, pulse timing was such that iTBS pulse triplets occurred around the tACS (anode) waveform peaks (with the middle pulse of each triplet occurring at the peak), tACS continued for the 3.3 minutes of the iTBS cycle, then faded off over 2 seconds. We confirmed via EEG that synchrony was maintained over the whole stimulation block to within 3 milliseconds (i.e., 1/67^th^, or 5°, of a theta cycle). In sham tACS conditions, pulse timing was such that iTBS pulse triplets initially occurred around tACS (anode) waveform troughs; however, the tACS faded off over 2 seconds under the cover of the first set of iTBS pulse triplets.

### 2.4 Electroencephalography (EEG)

#### 2.4.1 Data acquisition

58-channel EEG recordings were obtained with Brain Products actiCAP slim electrodes mounted within an appropriately sized actiCAP snap cap (extended international 10–20 arrangement). Electrodes and holders around the TMS location (F3, F1, F5, FC3, AF3) were removed to facilitate close approximation of the TMS coil to the scalp (TP9 was also removed as it overlay the tACS cathode). Signals were online referenced to FCz, with ground at Fpz, amplified with a Brain Products actiCHamp Plus system, online filtered between 0.1 and 400 Hz, sampled at 1 kHz, and stored for offline analysis.

#### 2.4.2 Resting-state and n-back tasks

All task procedures were implemented in PsychoPy (v2022.2.5), which sent event triggers to the EEG amplifier for storage alongside the EEG recordings. Tasks were presented on a 50 cm width monitor at an approximate distance of 55 cm. In resting-state blocks, participants closed their eyes for 100 s, then opened them, and viewed a fixation cross on a grey background for 100 s. An audio cue instructed them at each step. The n-back task resembled that used by Chung et al. (2019) in a study that identified participants’ spontaneous theta frequencies (for later personalisation of iTBS parameters). In n-back blocks, 500-ms stimuli (capital letters “A” to “J”) were presented one at a time in the centre of the screen, interspersed by 1-s fixation periods. Participants pressed a button as soon as they saw a letter that was the same as the letter two letters before (“two-back”)—both speed and accuracy were emphasised. After the first two stimuli, the target probability on each trial was 25%, with the constraint that targets were not allowed to occur consecutively. 130 trials were presented during each n-back run.

#### 2.4.3 Data pre-processing

Data were pre-processed using the EEGLAB toolbox v2022.1 (Delorme & Makeig, 2004), which runs under Matlab (R2022b, The Mathworks, Natick, MA). Data were first band-pass filtered between 0.5 and 40 Hz, then bad channels were identified and removed (kurtosis limits ±5 SD; mean ± SD of 8 ± 4 channels). The first 10 seconds (and last 5 seconds) of each resting-state period were excluded as participants were responding to the cue to open or close their eyes during this window. Resting-state data were split into 3-second non-overlapping epochs, and task data were split into 3-second overlapping epochs around non-target stimuli. Epochs containing extreme values (joint probability limits ±3.5 SD) were removed in each case (mean ± SD of 15 ± 7% and 17 ± 7% epochs rejected for rest and task data). Then, resting-state and alternate (to ensure non-overlapping) task data epochs were concatenated and submitted to independent component analysis (ICA) using the Infomax extended algorithm (Bell & Sejnowski, 1995; Lee et al., 1999; Makeig et al., 1995) accelerated with the CUDAICA toolbox (Raimondo et al., 2012) (ICA performed on mean ± SD of 338 ± 29 epochs, i.e., 17 minutes of data). Components representing clear sources of noise (eye blinks, lateral eye movements, single-channel noise) were identified from their scalp topographies and time courses and removed. ICA component weights and rejections were then applied to the full overlapping set of non-target task data epochs. Data were re-referenced to average reference, then Morlet wavelet time-frequency decomposition was computed at 1-Hz intervals from 1–30 Hz using the mfeeg toolbox (Wu et al., 2015). For task data, this was conducted for the frontal midline AFz and Fz electrodes, and mean power across the electrodes at theta (4–8 Hz) frequencies within the 1-second inter-stimulus fixation periods following non-target trials (excluding the small number containing an incorrect response) was extracted for each measurement time point and stimulation condition. We focus on non-target trials since targets could contain brain activity related to motor responses. We focus on inter-stimulus periods because, even though fronto-central theta would be expected to be elevated during all phases of the n-back task (since stimuli, and their temporal order, must be held in memory throughout), theta is typically largest during the inter-stimulus (memory maintenance) phase. Since our interest was not on modulations of theta power within a trial, but on estimating changes in maximal theta following stimulation, we expressed task frontal theta power at each post-stimulation time point in decibels relative to pre-stimulation task frontal theta power. As our primary hypotheses did not concern resting-state, we ran exploratory analyses for that dataset, with power also extracted for beta (13–30 Hz) frequencies at the Cz (central midline) electrode, and alpha (8–13 Hz) frequencies at the Oz (central occipital) electrode (electrodes at which these frequency bands tend to be maximal). Similar to task data, resting power for each post-stimulation time point, frequency band, and condition (eyes open or closed) was expressed in decibels relative to pre-stimulation resting power for that frequency band and condition.

### 2.5 Emotional bias task

#### 2.5.1 Task description

Twenty minutes post-stimulation, after the end of the EEG measurements, participants completed two variants of the CANTAB emotional bias task (Cambridge Cognition, Cambridge, UK), presented on a compatible iPad (Apple, Cupertino, CA). This standardised task contains a brief explanation, followed by a series of faces morphed between two emotions of varied intensities. In the first variant, the emotions were happy and sad, in the second they were happy and disgusted. Faces were displayed for 150 ms followed by a black and white noise mask. The participant then pressed a label to indicate the perceived emotion, after which a central fixation cross appeared followed by the next face. Each variant contained two practice trials and 45 assessed trials. The primary outcome measure from each variant is the “bias point,” which corresponds to the proportion of faces reported as “happy” scaled to have a value between 0 and 15, where 7.5 represents equal responding happy and the relevant negative emotion.

### 2.6 Statistical analysis

#### 2.6.1 Statistical analysis

Data were analysed using mixed-effects models implemented in SPSS (v28.0.1.1, IBM, Armonk, NY) and estimated with restricted maximum likelihood. Analyses used all available data for each participant. Participants served as a random effect with a scaled identity variance-covariance matrix. All analyses were two-tailed, with a significance threshold of 0.05. Post-hoc exploration of significant terms applied false discovery rate correction (Benjamini & Hochberg, 1995; Benjamini & Yekutieli, 2001) using the implementation from EEGLAB (Delorme & Makeig, 2004). For the EEG analyses, fixed effects were post-stimulation time point (1, 8, and 15-minutes post-stimulation) and stimulation condition (tsiTBS, iTBS+sham, tACS+sham, double sham). All statistical models incorporated the interaction effect between time point and stimulation condition. For the primary task EEG analyses, only one mixed-effects model was run (on frontal theta power). For the secondary resting-state EEG analyses, mixed-effects models were run for frontal theta power, as well as separate, exploratory, mixed-effects models for central beta power, and occipital alpha power (in each case, for the eyes open and eyes closed conditions separately). For task behavioural analyses, two models were run for accuracy (percent of responses to targets or non-targets) and one for mean target reaction time (with fixed factors of stimulation condition, and time point—including pre-stimulation and 1-, 8-, and 15-minutes post-stimulation). For the emotional bias task data, one mixed-effects model was run (on emotional bias point), and fixed effects were stimulation condition and task variant (happy-sad or happy-disgust). As control analyses, we also ran each mixed-effects model with session number replacing stimulation condition (to determine whether differences were due to session order or repetition, rather than due to differences in stimulation). There were no significant effects involving the session number term in any case. In separate control analyses, we ran each mixed model including an additional dummy regressor (alongside stimulation condition and timepoint). The dummy variable was coded as one for the first session, and zero for the other sessions. There were no significant effects involving the dummy regressor, and other reported effects remained significant.

## 3 Results

### 3.1 Task performance

Overall, for the n-back task, responses were correctly made to 76.8 ± 15.2 (mean ± SD) percent of targets (Table 1), and responses were correctly withheld to 97.3 ± 2.7 percent of non-targets. Neither percentage varied significantly across stimulation conditions or post-stimulation time points, nor did D-prime (d’), a signal detection measure that combines the hit (true positive) and false alarm (false positive) rates (mean d’ 2.8 ± 0.7; main effects and interactions: *p* > 0.1). Mean reaction time to targets was 650 ± 111 milliseconds. This too did not vary significantly across conditions or time points (*p* > 0.1).

**Table 1.** Percentage of targets correctly responded to (mean ± SD) at each n-back time point for each of the four stimulation conditions.

	Pre-stimulation	+1 minute	+8 minutes	+15 minutes
Sham+Sham	78.6 ± 10.3%	74.7 ± 14.9%	75.3 ± 17.3%	73.1 ± 14.2%
tsiTBS	77.4 ± 12.1%	78.7 ± 11.8%	76.0 ± 17.5%	73.5 ± 19.2%
iTBS+Sham	80.4 ± 16.7%	78.1 ± 16.6%	77.0 ± 18.6%	77.9 ± 16.4%
tACS+Sham	76.3 ± 18.9%	75.9 ± 14.8%	76.3 ± 11.3%	79.2 ± 11.8%

### 3.2 Task-related frontal theta oscillatory power

**Fig. 2.**
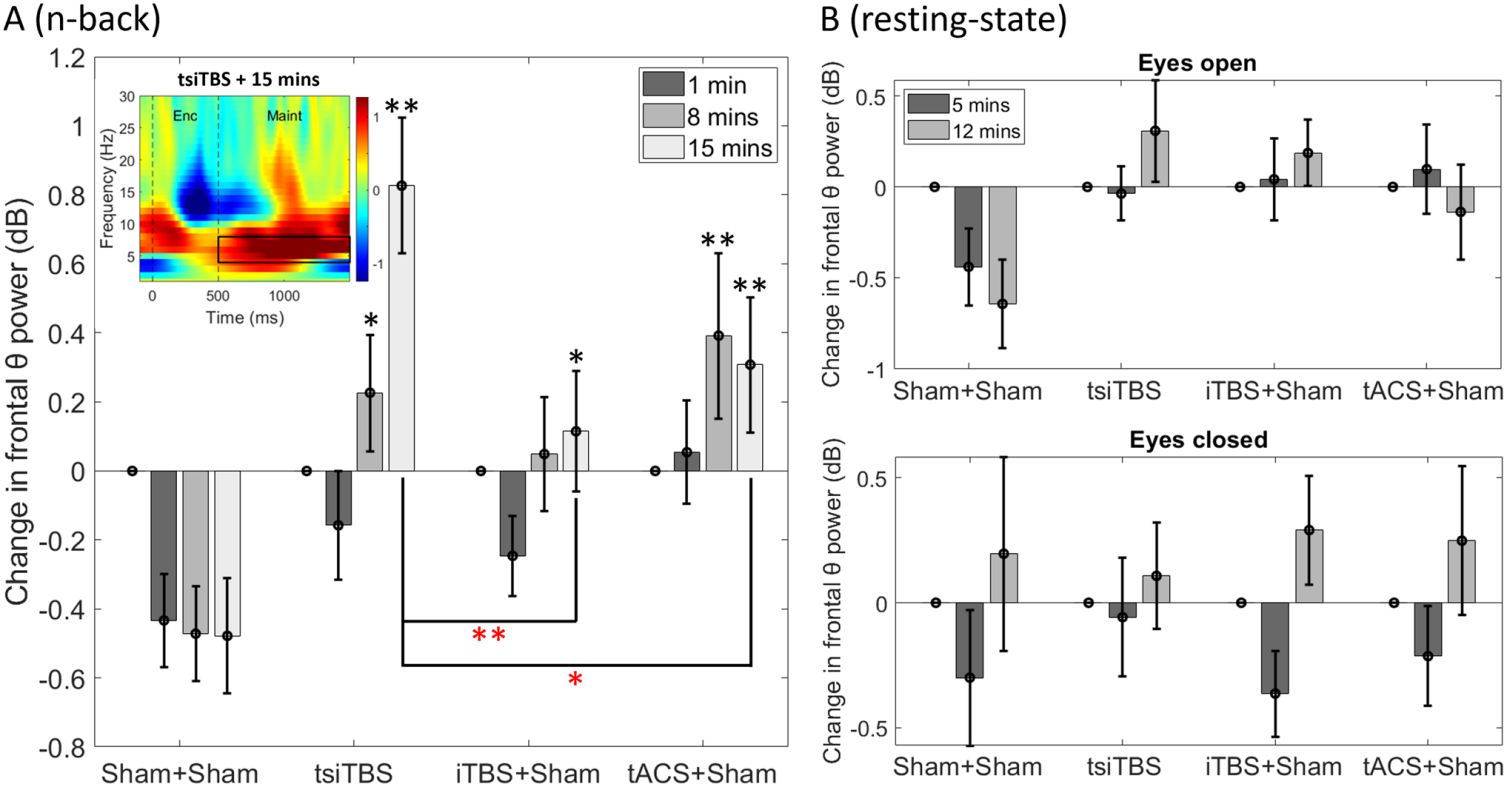
Change in frontal theta power across post-stimulation time points, measured during the maintenance (inter-stimulus) phase of an n-back working memory task (A), or at rest (B). Values are decibels relative to pre-stimulation theta power. Error bars are ±1 S.E.M. In (A), black asterisks above bars indicate significance compared to the corresponding time point in double sham (*p < 0.05, **p < 0.01, FDR-corrected); red asterisks with lines indicate significance compared to the corresponding time point in other stimulation conditions. Significant differences are not illustrated in (B). Inset shows the group time-frequency spectrogram measured 15 minutes after tsiTBS during the memory task (red = power increases, blue = power decreases, relative to mean pre-stimulation power at each frequency). Enc: memory encoding phase of task (letter display), Maint: maintenance phase of task (inter-stimulus period). The black rectangle shows the time and frequency window used for extracting theta power.

Figure 2A shows change in frontal theta power measured during the n-back task 1-, 8-, and 15 minutes post-stimulation (relative to pre-stimulation measurements). Theta power dropped following double sham (i.e., with task repetition within the session). This drop was reduced in the three active stimulation conditions. Following tsiTBS, theta rose steeply at the 8- and 15-minute time points and did not plateau. The rise was smaller following TMS alone. Following tACS alone, the initial rise in theta up to the 8-minute time point was numerically greater than following tsiTBS, but appeared less sustained, with theta falling by the 15-minute time point.

In a linear mixed-model analysis, the main effects of stimulation condition [*F*(3,204.070) = 11.026, *p* < 0.001] and post-stimulation time point [*F*(2,193.322) = 6.537, *p* = 0.002] on theta change were both significant, as was the interaction of stimulation condition and post-stimulation time point [*F*(6,193.322) = 2.363, *p* = 0.032].

Univariate tests indicated that theta change differed across stimulation conditions at 8 minutes [*F*(3,197.354) = 4.377, *p *= 0.005] and 15 minutes [*F*(3,197.354) = 10.690, *p* < 0.001], but not at 1 minute [*F*(3,197.354) = 1.035, *p* = 0.378], post-stimulation. We therefore conducted follow-up pair-wise tests between stimulation conditions at the two latter time points, applying false discovery rate correction across pairs. At 8 minutes, theta change in both the tsiTBS and tACS only conditions was greater than double sham (adjusted *p* = 0.015 and 0.006). Theta change in the TMS-only condition approached significance from double sham (adjusted *p* = 0.066). The active conditions did not differ from one another (adjusted *p* = 1). At 15 minutes, theta change in all active conditions was greater than double sham (adjusted *p* = 0.006, 0.018, and 0.006, for the tsiTBS, TMS-only, and tACS-only conditions). In addition, theta change was greater in the tsiTBS condition than either the TMS-only (adjusted *p* = 0.006) or tACS-only (adjusted *p* = 0.018) conditions. The TMS-only and tACS-only conditions did not differ significantly from each other (adjusted *p* = 1).

### 3.3 Resting-state oscillatory power

Figure 2B shows change in resting-state frontal theta power measured 5 and 12 minutes post-stimulation in the eyes open (top) and closed (bottom) conditions (relative to pre-stimulation measurements). Numerically, changes in theta power from the resting-state eyes open measurements mimicked those from the n-back measurements. However, there was a significant main effect of stimulation condition only [*F*(3,126.320) = 4.254, *p* = 0.007; main effect of time point: *F*(1,118.520) = 0.009, *p* = 0.926; interaction: *F*(3,118.520) = 1.042, *p* = 0.377]. Theta change was greater for either the tsiTBS (adjusted *p* = 0.009) or TMS-only (adjusted *p* = 0.009) conditions than double sham. Theta change in the tACS-only condition did not differ significantly from double sham (adjusted *p* = 0.088), nor did the stimulation conditions differ from one another (adjusted *p* = 1). For the eyes closed measurements, there was only a significant main effect of time point [*F*(1,123.039) = 8.060, *p* = 0.005; main effect of condition: *F*(3,130.044) = 0.030, *p* = 0.993; interaction: *F*(3,123.039) = 0.453, *p* = 0.716]—theta power initially decreased, then rose to above baseline levels. Resting-state analyses were also conducted for central beta, and occipital alpha, power. There were no significant effects for either eyes open or closed measurements (*p* > 0.1).

### 3.4 Emotional bias

**Fig. 3.**
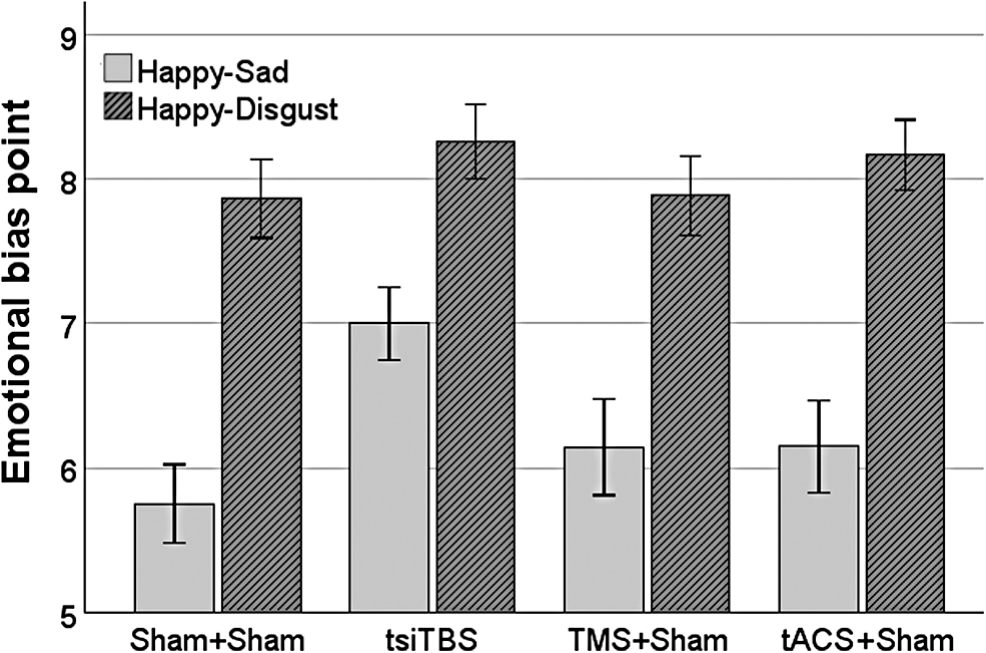
Emotional bias points for the two task variants, measured 20 minutes after each of the four types of stimulation. Error bars are ±1 S.E.M. Higher emotional bias point corresponds to a greater probability of reporting a presented face as conveying a happy emotion (rather than a sad or disgusted emotion). Significant differences were observed between tsiTBS and each of the other stimulation conditions, collapsing across emotion (not shown).

Figure 3 shows emotional bias measured 20 minutes post-stimulation following each of the stimulation conditions, for the happy-sad and happy-disgusted task variants. In each case, higher values of emotional bias indicate greater reporting of emotions as “happy.” There were significant main effects of stimulation condition [*F*(3,137.416) = 5.236, *p* = 0.002] and task variant [*F*(1,131.714) = 141.306, *p* < 0.001], but no significant interaction [*F*(3,131.714) = 1.725, *p* = 0.165]. The effect of task variant relates to generally higher emotional bias values for the happy-disgusted, than happy-sad, variant. The effect of stimulation condition relates to higher (more positive) emotional bias values following tsiTBS than following double sham (adjusted *p* = 0.006), TMS alone (adjusted *p* = 0.009), or tACS alone (adjusted *p* = 0.024). To guide future studies, a fully independent-samples design would require a sample size of 33 per group to detect the observed difference in happy-sad emotional bias point between tsiTBS and TMS-alone groups (Cohen’s *d* = 0.623), whilst a fully repeated-measures design would require a sample size of 15 (*dz* = 0.699; one-tailed *t*-tests with 80% power, α = 0.05; G*Power v3.1.9.7).

### 3.5 Adverse effects

There were no serious adverse effects. In two sessions, headache was rated as “severe” (one in tsiTBS, one in TMS alone), and symptoms resolved within a minute of stimulation cessation. Percentage of sessions with at least one adverse effect rated as “moderate” was 25%, 17%, 19%, and 26% in the sham, tACS-only, TMS-only, and tsiTBS conditions. Including reports of “mild” symptoms, participants reported a mean of 2.0, 1.7, 1.3, and 2.0 adverse effects in the sham, tACS-only, TMS-only, and tsiTBS conditions [main effect of stimulation type on number of reported symptoms: *F*(3,57.326) = 1.221, *p* = 0.310]. Overall, the most reported adverse effects were headache (51% of sessions), drowsiness (39%), and scalp discomfort (30%). The remainder of adverse effects listed were reported on fewer than 15% of sessions.

## 4 Discussion

### 4.1 Summary of findings

As predicted, frontal theta oscillatory power, measured during the n-back task, increased to a greater extent following tsiTBS than following double sham or either stimulation method alone. Theta increased markedly between the eight- and 15-minute post-stimulation time points, and thus may still have not reached its peak value during our measurements. On the other hand, there was indication that the theta increase following tACS alone peaked earlier, around 8 minutes post-stimulation. As predicted, emotional bias, measured 20 minutes post-stimulation using a previously validated commercial task, was more positive following tsiTBS than following double sham or either stimulation method alone.

### 4.2 Changes in theta power following stimulation

Hosseinian, Yavari, Kuo, et al. (2021) delivered 20 minutes of 6 Hz (non-iTBS) rTMS with synchronised 6 Hz tACS. Frontal theta measured during an n-back task shortly after stimulation cessation was 28% (1.07 dB) or 46% (1.64 dB) greater than pre-stimulation measurements (one- or three-back task). This could be entirely accounted for by the effects of tACS alone, whilst there was little change following rTMS alone. In contrast, we observed, following 3 minutes of iTBS with synchronised 5 Hz tACS (tsiTBS), an initial drop in n-back theta power that rose 0.83 dB above baseline 15 minutes post-stimulation. This was significantly greater than the rise following tACS alone (peaking at 0.39 dB), although we too found little theta increase above baseline following iTBS alone.

Whilst there is a difference in stimulation type (iTBS versus non-iTBS, 5 Hz versus 6 Hz, unilateral versus bilateral stimulation) between ours and Hosseinian et al.’s studies, we suggest that qualitative differences may relate to the durations of stimulation, measurement time points, and, importantly, the time courses of the effects of stimulation on oscillatory activity. Specifically, it may be that stimulation does not directly enhance theta activity but instead induces a process that manifests as theta enhancement after several minutes. Whilst the degree and duration of the enhancement might be modified by stimulation duration, the build-up of theta may continue whether stimulation is ongoing or has ceased. TMS+tACS may serve to make the enhancement longer lasting than tACS or TMS alone. The initial build-up of theta would have been missed by Hosseinian et al. since their first measurements were 20 minutes after the start of stimulation. The absence of difference between their TMS+tACS and tACS-only conditions may be because they measured n-back theta at a single time point. They did record resting-state theta across an hour post-stimulation. Eyes open resting-state frontal theta was 20% (0.79 dB) greater immediately after the cessation of combined stimulation, a level that only fell after 50 minutes. tACS alone led to a lower-level rise (12%, 0.49 dB), but importantly also fell earlier, 30 minutes after stimulation.

Maiella et al. (2022) reported increases in resting-state gamma, but not theta, power following iTBS with synchronised gamma tACS, but did not find gamma or theta changes with the use of synchronised theta-tACS or with sham tACS. Our results suggest that the absence of theta effects was due to measurement too soon after stimulation cessation. In addition, we observed more robust effects with the use of an n-back task. Thus, a short tsiTBS sequence can boost theta power, an effect that accrues across at least 15–20 minutes since stimulation onset.

Finally, we did not observe changes in performance on the n-back task itself. In contrast, Hosseinian et al. found improved performance following combined stimulation on the three-back, but not the one-back, task. It may have been that observing performance changes requires the more demanding task. Alternatively, it is possible that improvements in cognition require longer stimulation durations (despite short durations being sufficient to enhance frontal theta per se). Studies reporting improvements in working memory following tACS have used at least 10-minute stimulation periods (Nissim et al., 2023). A recent study found a reduction in reaction time on a two-back working memory task following 8 minutes of iTBS (Razza et al., 2023). Shorter iTBS protocols have yielded some significant, although typically small or inconsistent, improvements (Lowe et al., 2018; Viejo-Sobera et al., 2017).

### 4.3 Impact of tsiTBS on emotional bias

We observed significantly more positive emotional bias 20 minutes after tsiTBS than after double sham or either stimulation method alone. We included these measurements as a marker of potential antidepressant efficacy (Cotter & Barnett, 2018; Harmer & Cowen, 2013). Negative biases in MDD contribute to the persistence of symptoms (Roiser et al., 2012) but normalise following successful treatment (Ruohonen et al., 2020). Early shifts in emotional bias may predict treatment outcomes (Warren et al., 2015), and generally effective antidepressant medications modify emotional biases after one dose, including in non-patients (Harmer et al., 2003). The more positive emotional bias after tsiTBS than iTBS alone suggests that tsiTBS holds promise as a more efficacious antidepressant therapy.

However, it was our initial assumption that addition of synchronised tACS might improve antidepressant efficacy by enhancing theta power *during* iTBS. Yet, whilst we found more positive emotional bias following tsiTBS, theta measured immediately after (and thus, presumably, during) tsiTBS was not yet elevated above baseline. It may be, therefore, that synchronised tACS modified the effects of iTBS during stimulation in another manner. It has been suggested that tACS might “entrain” underlying cortical oscillations (cause them to align in time with the stimulation waveform) (Thut et al., 2011). Subsequent work has not found clear evidence for this in human studies (Veniero et al., 2015; Vossen et al., 2015), but single-unit recordings in nonhuman primates have shown spike-timing entrainment with tACS intensities achievable in humans with 1–2 mA current (with greater effects at higher intensities) (Johnson et al., 2020). Alternatively, the propagation of the effects of TMS has been shown to vary according to the phase of concurrently applied tACS (Fehér et al., 2017; Schilberg et al., 2018). Propagation of neuromodulatory effects is important, since clinical efficacy of rTMS is thought to be due to modulation of activity in limbic brain regions distal from the site of stimulation, such as the subgenual anterior cingulate cortex (Duprat et al., 2022; Fox et al., 2012). Synchronised tACS may thus enhance effect propagation and make such modulation more robust. Speculatively, the subsequent theta power rise might then be consequent to enhancement of the activities of these connected regions. For instance, there is evidence that, alongside pre-frontal cortex, anterior cingulate is a key generator of frontal theta oscillatory activity (Hsieh & Ranganath, 2014).

### 4.4 Limitations

Whilst tsiTBS led to greater modulation of theta oscillatory brain activity, and emotional bias, than either technique alone, it may be that concurrent delivery of the two techniques (rather than synchronous delivery) is sufficient. Whilst our synchronisation approach was selected to be simple to implement without additional hardware (facilitating transfer to the clinical setting), removing the requirement for synchronisation would further simplify the approach. We will examine the necessity of synchronisation in a future study. However, we chose to focus on the synchronous condition given that Hosseinian, Yavari, Biagi, et al. (2021) found substantial differences in modulation of alpha brain activity following alpha tACS+rTMS with TMS pulses delivered at anodal peaks versus troughs and given that B. Zrenner et al. (2020) and C. Zrenner et al. (2018) found that rTMS-induced plasticity in motor pathways, and rTMS-induced modulation of oscillatory activity in DLPFC, differed whether TMS pulses were delivered at the peaks or troughs of spontaneous brain oscillatory activity measured with concurrent EEG.

There is overlap between our approach, which delivers TMS pulses around the peaks of an imposed oscillation, and approaches that deliver TMS pulses at the peaks of spontaneous oscillations measured with EEG (B. Zrenner et al., 2019). Benefits of our approach include that it does not rely on rapid processing of noisy recordings, and is suitable for use with oscillatory frequencies, such as theta, which are typically weak at rest. (Vossen et al., 2015). Moreover, spontaneous oscillatory activity is abnormal in MDD (Fingelkurts & Fingelkurts, 2015). It may be that a combined approach, in which tsiTBS is used to boost endogenous theta activity and initiate therapeutic neuroplastic processes, then EEG-triggered rTMS is used to continue and complete treatment, may be beneficial.

Further limitations of our study include that the tACS-only condition was introduced after the first two participants (as our original focus was on the addition of tACS to iTBS) and that only half of participants completed all four stimulation sessions. We ran two sets of control analyses without indication that order effects impacted our results (Section 2.6.1), but we cannot completely rule out more complex and unforeseen effects of order. The reduced ability to account for within-subject variance will have also lowered statistical power. Another limitation is that, whilst we measured change in theta power from baseline to follow-up within each experimental session, we only measured emotional bias post-stimulation. This matches the approach of the seminal studies on emotional bias following single doses of antidepressant medications which relied, as we do, on comparisons with emotional bias after placebo/sham (e.g., Browning et al., 2007; Harmer et al., 2003). Finally, as in Hosseinian, Yavari, Biagi et al. (2021) and Hosseinian, Yavari, Kuo, et al. (2021), the presence of the tACS electrodes increased the distance from the TMS coil to the cortex. We suggest, therefore, that our iTBS was received at approximately 80% of “active” rather than resting motor threshold (see Section 2.3.1). Further imprecision is created by our use of visually observed rMT, rather than rMT measured with motor evoked potentials. Eighty percent of active motor threshold is the traditional approach used for iTBS in MDD, although 80% of the higher, resting, motor threshold is often used in current practice (Chu et al., 2021). The relationship between motor threshold and optimal intensity for stimulation of DLPFC is unclear and possibly non-linear (Chung et al., 2018). Our findings should be explored at different stimulation intensities, therefore, as should the clinical effects of rTMS treatment themselves.

We did not ask participants to state which treatment they believed they received after each session, to avoid drawing their focus towards any differences in sensation. Therefore, we cannot quantify effects of expectation on our results. Anecdotally, any guesses showed little relation to the treatment given. We speculate this may be related to our relatively high incidence of mild-moderate adverse effects, present to a similar extent in the tsiTBS condition as in the double sham condition. Some of these may have been due to the testing conditions (dark room), tightness of the EEG cap, or pressure exerted by the EEG cap on the underlying tACS electrode. Four participants spontaneously reported that scalp discomfort or headache resolved immediately on removing the EEG cap. It should be noted, however, that no new safety concerns arose, consistent with findings from the small amount of literature on the safety of combined tACS and rTMS (Rossi et al., 2021). Few basic science studies report adverse effects in detail, but including these is important to guide risk/benefit discussions in future clinical studies with patients.

## 5 Conclusions

We found that tsiTBS leads to subsequent enhancement of frontal theta oscillatory brain activity, building over at least 15 minutes post-stimulation. It is also associated with a more positive bias in reporting facial emotions, a previously proposed marker of antidepressant efficacy. Both these effects were greater than those following iTBS alone. Thus, tsiTBS holds promise as an alternative neuromodulation therapy for MDD, or, potentially, as an augmentation strategy when rTMS alone is not sufficiently effective. Confirmation will depend on future, multi-session, studies with people with MDD. Further work is needed to understand the mechanisms by which synchronised tACS augments the neuromodulatory effects of iTBS.

## Data Availability

The datasets used in the current study are available from the corresponding author on completion of a formal data sharing agreement. They are part of an ongoing study and will be made publicly available at a later date.
